# Al exposure increases proline levels by different pathways in an Al-sensitive and an Al-tolerant rye genotype

**DOI:** 10.1038/s41598-020-73358-9

**Published:** 2020-10-02

**Authors:** Alexandra de Sousa, Hamada AbdElgawad, Fernanda Fidalgo, Jorge Teixeira, Manuela Matos, Badreldin A. Hamed, Samy Selim, Wael N. Hozzein, Gerrit T. S. Beemster, Han Asard

**Affiliations:** 1 Plant Stress Lab - GreenUPorto Sustainable Agrifood Production Research Center, Biology Department, Faculty of Sciences, University of Porto, Rua do Campo Alegre s/n, 4169-007 Porto, Portugal; 2 Laboratory for Integrated Molecular Plant Physiology Research (IMPRES), Department of Biology, University of Antwerp, 2020 Antwerp, Belgium; 3 Biosystems and Integrative Sciences Institute (BioISI), Department of Genetics and Biotechnology, UTAD- University of Trás-Os-Montes E Alto-Douro, Quinta dos Prados, 5000-801 Vila Real, Portugal; 4 Botany and Microbiology Department, Faculty of Science, Beni-Suef University, Beni-Suef, 62511 Egypt; 5 Microbiology and Botany Department, Faculty of Science, Suez Canal University, Ismailia, 41522 Egypt; 6 Zoology Department, College of Science, Bioproducts Research Chair, King Saud University, Riyadh, 11451 Saudi Arabia

**Keywords:** Plant stress responses, Plant sciences, Environmental sciences

## Abstract

Aluminium (Al) toxicity limits crop productivity, particularly at low soil pH. Proline (Pro) plays a role in protecting plants against various abiotic stresses. Using the relatively Al-tolerant cereal rye (*Secale cereale* L.), we evaluated Pro metabolism in roots and shoots of two genotypes differing in Al tolerance, var. RioDeva (sensitive) and var. Beira (tolerant). Most enzyme activities and metabolites of Pro biosynthesis were analysed. Al induced increases in Pro levels in each genotype, but the mechanisms were different and were also different between roots and shoots. The Al-tolerant genotype accumulated highest Pro levels and this stronger increase was ascribed to simultaneous activation of the ornithine (Orn)-biosynthetic pathway and decrease in Pro oxidation. The Orn pathway was particularly enhanced in roots. Nitrate reductase (NR) activity, N levels, and N/C ratios demonstrate that N-metabolism is less inhibited in the Al-tolerant line. The correlation between Pro changes and differences in Al-sensitivity between these two genotypes, supports a role for Pro in Al tolerance. Our results suggest that differential responses in Pro biosynthesis may be linked to N-availability. Understanding the role of Pro in differences between genotypes in stress responses, could be valuable in plant selection and breeding for Al resistance.

## Introduction

Proline (Pro) is involved in a wide range of plant physiological and developmental processes^1^. In addition to being a proteinogenic amino acid, Pro contributes to stress-mitigation, as a compatible solute for osmotic adjustment, as an ROS scavenger and as a molecular chaperone stabilizing proteins and membranes. Moreover, changes in Pro metabolism may affect the cellular redox status, prompting for metabolic adjustments^2^. Pro can also contribute to buffering cytosolic pH and can act as a source of energy, carbon, and nitrogen to support plant growth after stress relief^1,3^.

**Figure 1:**
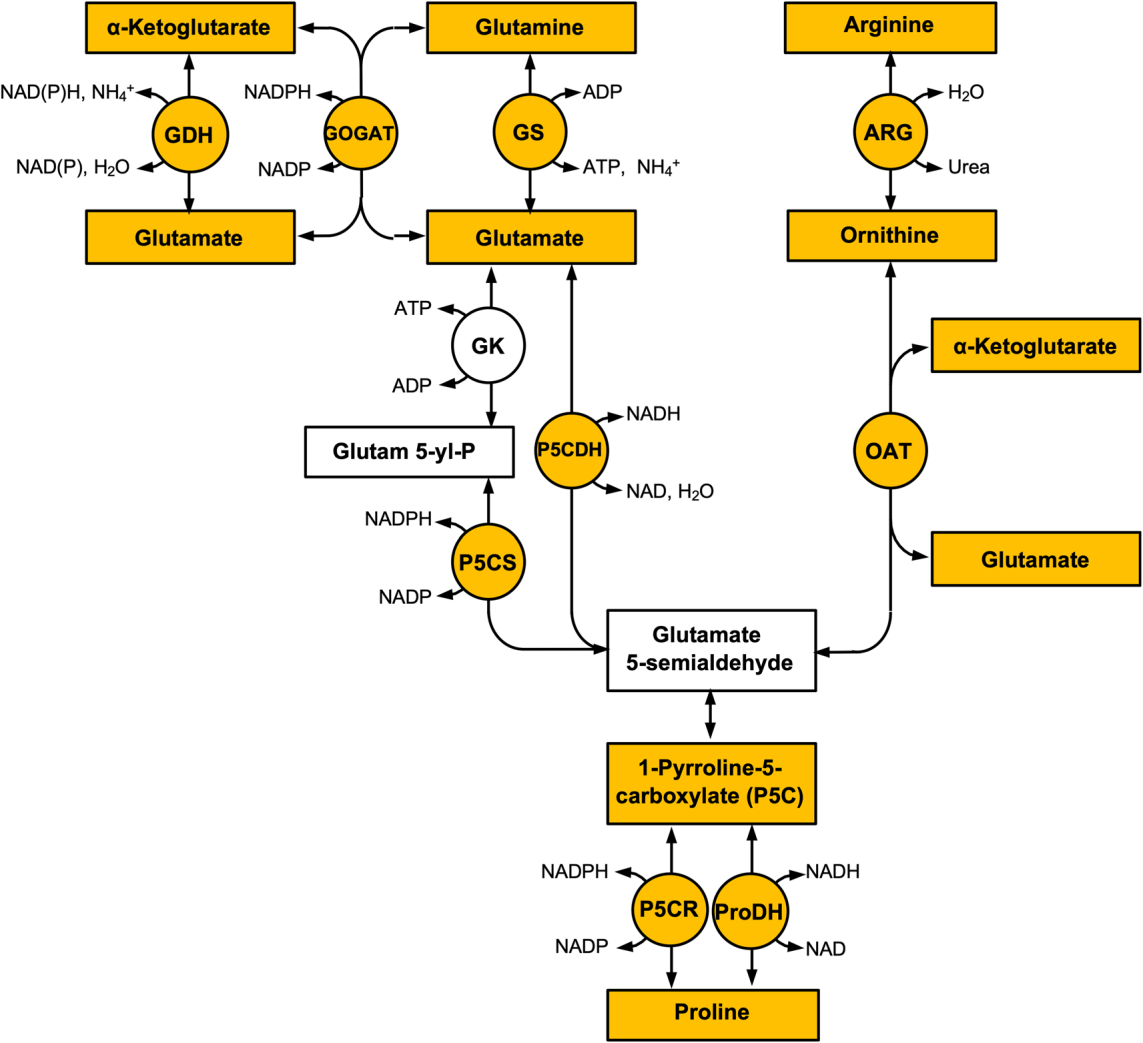
Scheme showing the Pro biosynthesis pathways metabolites (rectangles) and enzymes (circles). Coloured items are quantified in this study. Enzyme abbreviations: *ARG* arginase; *GDH* glutamate dehydrogenase; *GK* glutamate kinase; *GOGAT* glutamine oxoglutarate aminotransferase; *GS* glutamine synthetase; *OAT* ornithine aminotransferase; *ProDH* proline dehydrogenase; *P5CDH* pyrroline-5-carboxylate dehydrogenase; *P5CR* pyrroline-5-carboxylate reductase; and *P5CS* pyrroline-5-carboxylate synthase.

Despite the frequently demonstrated importance of Pro in plant functions, comparatively little is known on how exactly Pro levels are modified. Pro is not normally accumulated and stored, and therefore changes in Pro occur through changes in biosynthesis and re-oxidation^1^. The Pro pool is controlled by biosynthesis through (1) a glutamate (Glu)-dependent pathway, (2) an ornithine (Orn)-dependent pathway, and (3) by re-oxidation to 1-pyrroline-5-carboxylate (P5C)^4^ (Fig. 1). In studies in which changes in Pro-metabolic enzymes and metabolites were studied in detail, pathway-specific responses were observed. For example, Pro accumulation in rice exposed to Cu is enhanced through the Orn pathway^5^. However, in tobacco plants exposed to Cu and water deficit, Pro accumulation is enhanced through both the Glu and Orn pathways, as well as by reduced proline dehydrogenase activity (ProDH)^6^. The latter enzyme is generally considered to re-oxidize Pro. Additionally, increases in pyrroline-5-carboxylate synthase (P5CS) and ornithine aminotransferase (OAT) activities in peach exposed to cold stress suggests the involvement of both pathways^7^. Overall it appears that regulation of Pro synthesis may vary between species, and is based on stress-type, and duration.

As increased Pro levels may contribute considerably to plant stress tolerance, Pro is a worthwhile target for plant-resistance improvement through breeding, selection or genetic modification^8^. Therefore, a better understanding of how Pro levels are controlled in stress conditions is needed. Not only is rye an essential food source worldwide, it is also known for its relatively high Al tolerance^9^. Rye is therefore an interesting species to understand mechanisms to cope with Al toxicity. To help in this understanding, we compared responses in an Al-tolerant (var. Beira) and an Al-sensitive (var. RioDeva) rye genotype. Genotype-specificity is a useful tool to help identify molecular players in stress responses.

Al toxicity is an increasing agricultural problem, in particular in acidic soils, *i.e.* at pH below 5.5, when phytotoxic Al^3+^ predominates in solution. Soil acidity is increasing worldwide, largely due to anthropogenic inputs, and this currently occurs mostly in tropical and subtropical regions^9,10^. As much as 50% of arable land is estimated to be impacted negatively by Al due to acidic soil^11^. The effect of Al on plant growth, and root development in particular, has frequently been studied. From this, several mechanisms contributing to these effects have been identified^9,10^. For example, it is well-established that high soil Al levels stimulates the exudation of organic acids from the roots of several plant species, and the genes and proteins involved are being identified^9,12^. Al tolerance mechanisms also include phenolic and polypeptide exudation, mucilage secretion, phosphate efflux, alkalinization of the rhizosphere and Al adsorption to the cell wall^9,12^. In addition, we and others, have recently shown that elevated levels of the antioxidants ascorbate and glutathione, and their related antioxidant enzymes, are important for Al-tolerance in rye^13^. Unlike some of these well characterized Al-tolerance mechanisms the role of Pro in tolerance to Al is less well unravelled. For example, a role in metal-toxicity for Pro has been documented^1^, however rarely have changes in Pro been traced to the contributing biochemical pathways (Fig. 1), and to changes in N metabolism. To contribute to this lack in understanding, we investigated in detail changes in Pro metabolism in rye (*Secale cereale* L.) exposed to Al.

## Results

**Figure 2:**
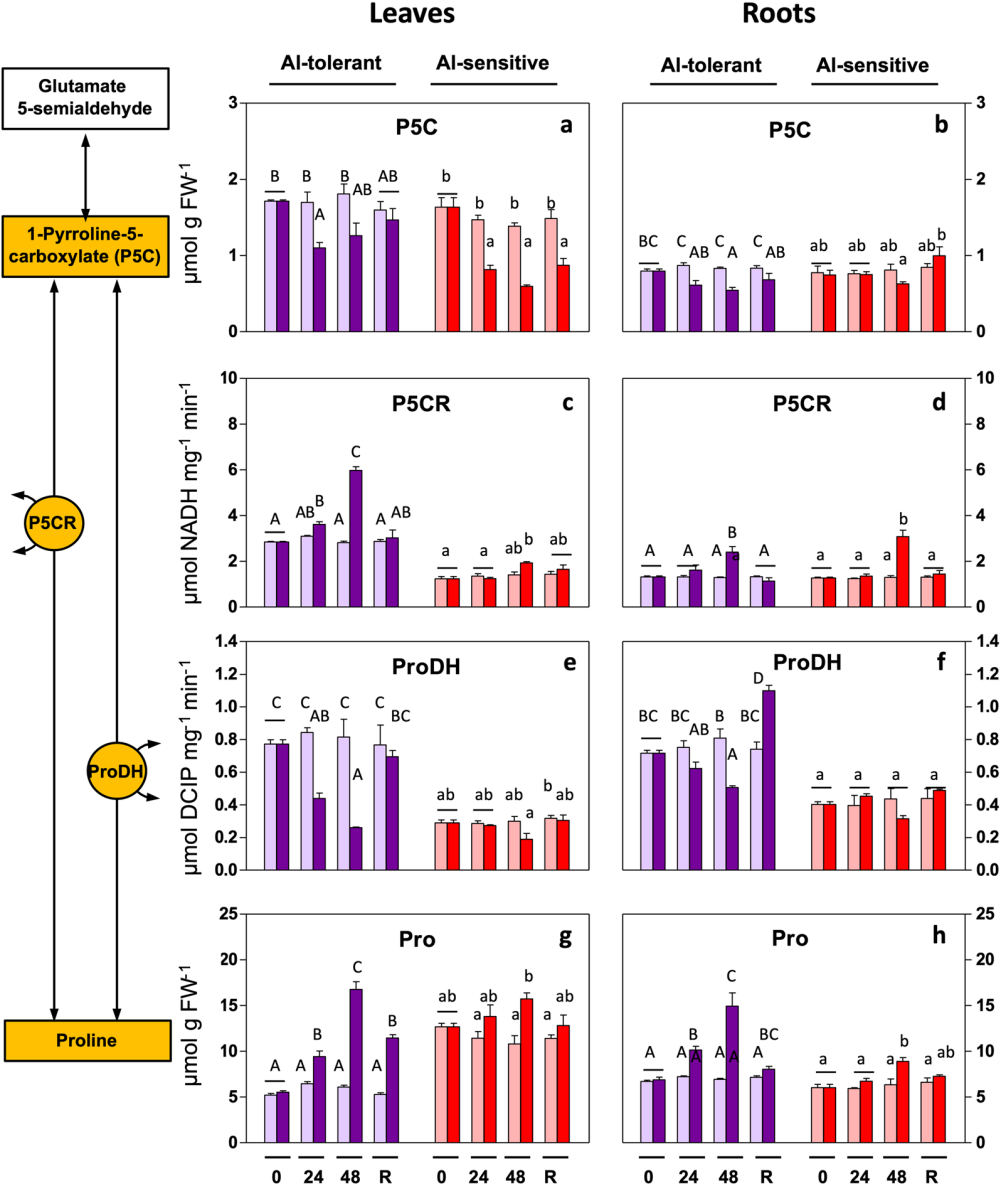
Al-induced changes in the conversion between P5C and Pro in rye seedlings. Metabolite levels and enzyme activities were determined in leaves and roots of Al-tolerant (Beira) and Al-sensitive (RioDeva) genotypes after 24 h and 48 h of exposure and 48 h of recovery. Bars: purple: genotype Beira; red: genotype RioDeva; light color: control (no Al); dark color: 5 mg L^−1^ Al. Panels: (**a**,**b**): pyrroline-5-carboxylate (P5C); (**c**,**d**): pyrroline-5-carboxylate reductase (P5CR); (**e**,**f**): proline dehydrogenase (ProDH); (**g**,**h**): proline (Pro). The results are the mean of 3 or 4 experiments (SD, see Methods). Letters indicate a significant difference at *P* < 0.05 (capital letters: tolerant line; small letters: sensitive line).

Pro levels are roughly similar in roots and leaves in non-stressed plants of the tolerant line (Fig. 2g,h). On the other hand, in the Al-sensitive line, Pro is approximately twofold higher in leaves than in roots. In leaves as well as in roots, Pro levels increased upon Al exposure, and this increase was statistically significant faster (at 24 h) in the tolerant genotype. The increase was higher with prolonged (48 h) exposure and was considerably stronger in the Al-tolerant line. Increases in Pro recovered to some extend to control levels 48 h after metal removal. Pro concentrations in plants are the result of two pathways synthesizing GSA, respectively through Glu and Orn, and Pro oxidation. To understand the mechanism by which Al induces Pro, we analysed metabolites and enzymes associated with each pathway (Fig. 1).

### Glu pathway for Pro synthesis

**Figure 3:**
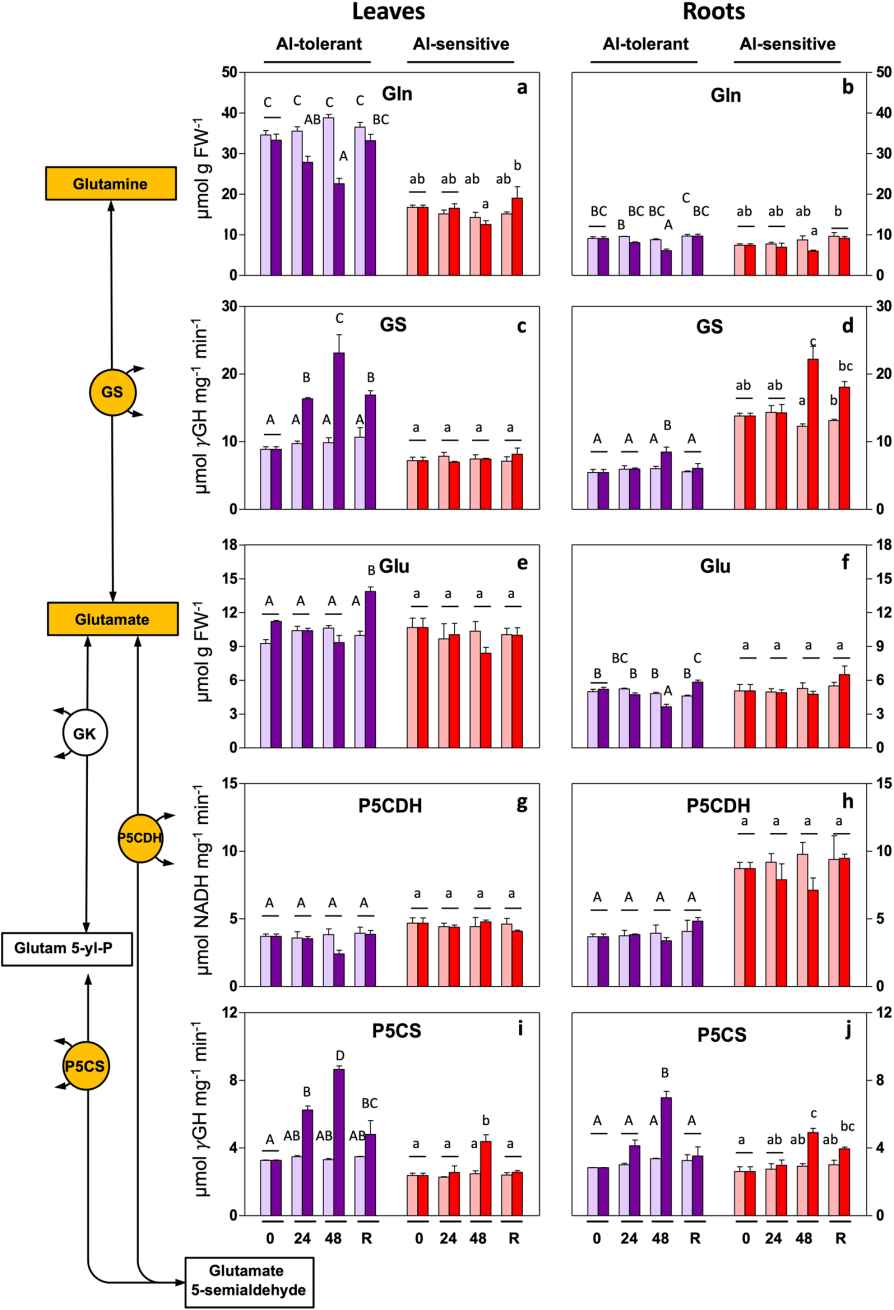
Al-induced changes in the Glu pathway for Pro biosynthesis in rye seedlings. Metabolite levels and enzyme activities were determined in leaves and roots of Al-tolerant (Beira) and Al-sensitive (RioDeva) genotypes after 24 h and 48 h of exposure and 48 h of recovery. Bars: purple: genotype Beira; red: genotype RioDeva; light color: control (no Al); dark color: 5 mg L^−1^ Al. Panels: (**a**,**b**): glutamine (Gln); (**c**,**d**): glutamine synthetase (GS); (**e**,**f)**: glutamate (Glu); (**g**,**h**): pyrroline-5-carboxylate dehydrogenase (P5CDH); (**i**,**j**): pyrroline-5-carboxylate synthase (P5CS). γGH: γ-glutamyl hydroxamate. The results are the mean of 3 or 4 experiments (SD, see Methods). Letters indicate a significant difference at *P* < 0.05 (capital letters: tolerant line; small letters: sensitive line).

Glu and Gln are essential substrates in this pathway. In leaves, Al stress reduced Gln in the tolerant genotype, but for Glu there were no significant changes (Fig. 3a,e). At the level of enzymes, in the leaves of the tolerant line, GS and P5CS activity increased, and P5CDH activity remained unchanged (Fig. 3c,g–i). In the sensitive line, on the other hand, GS and P5CDH were unaltered. P5CS increased in leaves at 48 h (Fig. 3i), but to a lesser extent than in the tolerant genotype (70% increase instead of 160% increase, at 48 h).

Under Al treatment, the Gln and Glu levels were reduced in roots of the Al-tolerant genotype and no changes were observed in the sensitive line (Fig. 3b,f). In roots, GS activity increased in both genotypes after 48 h, reaching higher values in the sensitive line (Fig. 3d). P5CS was induced in the roots of both genotypes (Fig. 3j), and P5CDH remained unchanged in both lines (Fig. 3h), as in leaves.

Al-induced changes in enzyme activities, in leaves and roots, generally, fully or partially recovered after removal of Al (Fig. 3). Together these results indicate differences in induction of the Glu pathway for Pro synthesis in the organs and genotypes studied.

### Orn pathway for Pro synthesis

**Figure 4:**
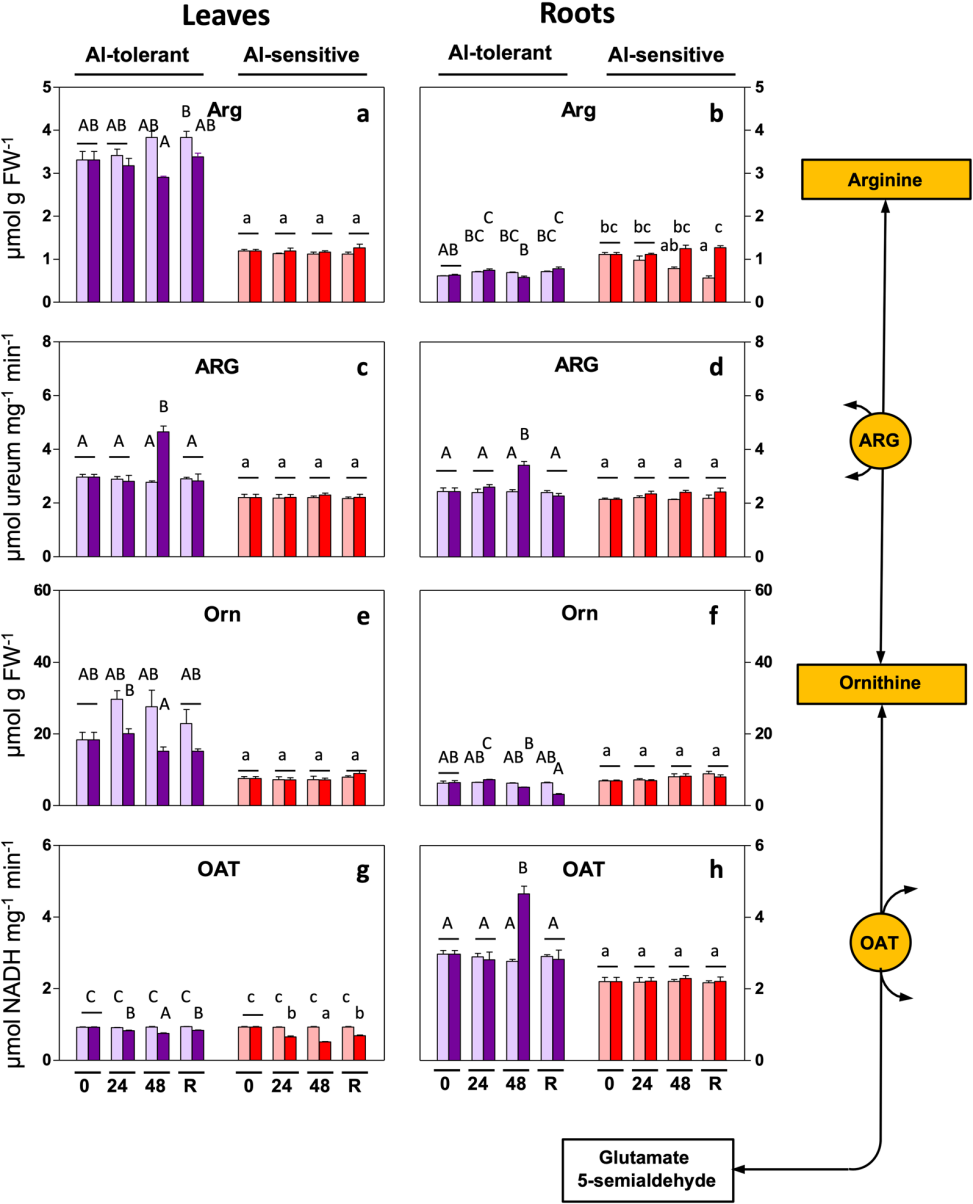
Al-induced changes in the Orn pathway for Pro biosynthesis in rye seedlings. Metabolite levels and enzyme activities were determined in leaves and roots of Al-tolerant (Beira) and Al-sensitive (RioDeva) genotypes after 24 h and 48 h of exposure and 48 h of recovery. Bars: purple: genotype Beira; red: genotype RioDeva; light color: control (no Al); dark color: 5 mg L^−1^ Al. Panels: (**a**,**b**): arginine (Arg); (**c**,**d**): arginase (ARG); (**e**,**f**): ornithine (Orn); (**g**,**h**): ornithine aminotransferase (OAT). The results are the mean of 3 or 4 experiments (SD, see Methods). Letters indicate a significant difference at *P* < 0.05 (capital letters: tolerant line; small letters: sensitive line).

For the Orn pathway, in the leaves, Al treatment had no significant effects on the substrates Arg and Orn in either genotype (Fig. 4a,e). Also in the leaves, ARG activity increased at 48 h of exposure in the Al-tolerant line, and OAT decreased in both lines (Fig. 4c,g).

In roots, Arg and Orn remained unchanged in both genotypes, under Al exposure (Fig. 4b,f). Activities of the enzymes ARG and OAT in roots increased in the tolerant line at 48 h after exposure but did not change in the sensitive line (Fig. 4d,h). Results of the Orn pathway also suggest changes specifically in the organs and genotypes investigated.

### Conversion of P5C to Pro

The common end-product of the Glu and Orn pathways, P5C, is converted to Pro, by P5CR. Another enzyme determining the Pro and P5C concentrations is ProDH, which generally re-oxidizes Pro. It is noteworthy that Pro levels, also in the absence of Al, were about twofold higher in the leaves of the sensitive line.

In leaves, the Al treatment reduced P5C levels (significant in the tolerant line at 24 h only) and induced Pro levels, in both genotypes (significant in the sensitive line at 48 h) (Fig. 2a). In roots under Al exposure, P5C levels decreased in the tolerant genotype (significant at 48 h), but not in the sensitive line. Pro levels increased significantly at 24 and 48 h in the tolerant line, and at 48 h in the sensitive line (Fig. 2g). The Pro increase was about twofold in the tolerant line, but only a 20% increase in the sensitive line was observed. Increases were only significant in the sensitive line after prolonged exposure (48 h) (Fig. 2b,h). P5CR activity increased in the Al-tolerant genotype, while ProDH activity decreased (Fig. 4c,e). No changes in enzyme activities of P5CR and ProDH were observed in leaves of the Al-sensitive genotype. P5CR activity in the roots increased only at 48 h after exposure (Fig. 2d).

ProDH activity decreased in the Al-tolerant genotype but remained unchanged in the sensitive line (Fig. 2f). Generally, both metabolite levels and enzyme activities, fully recovered, in leaves and roots of both genotypes, after Al removal.

### Nitrogen (N) assimilation

**Figure 5:**
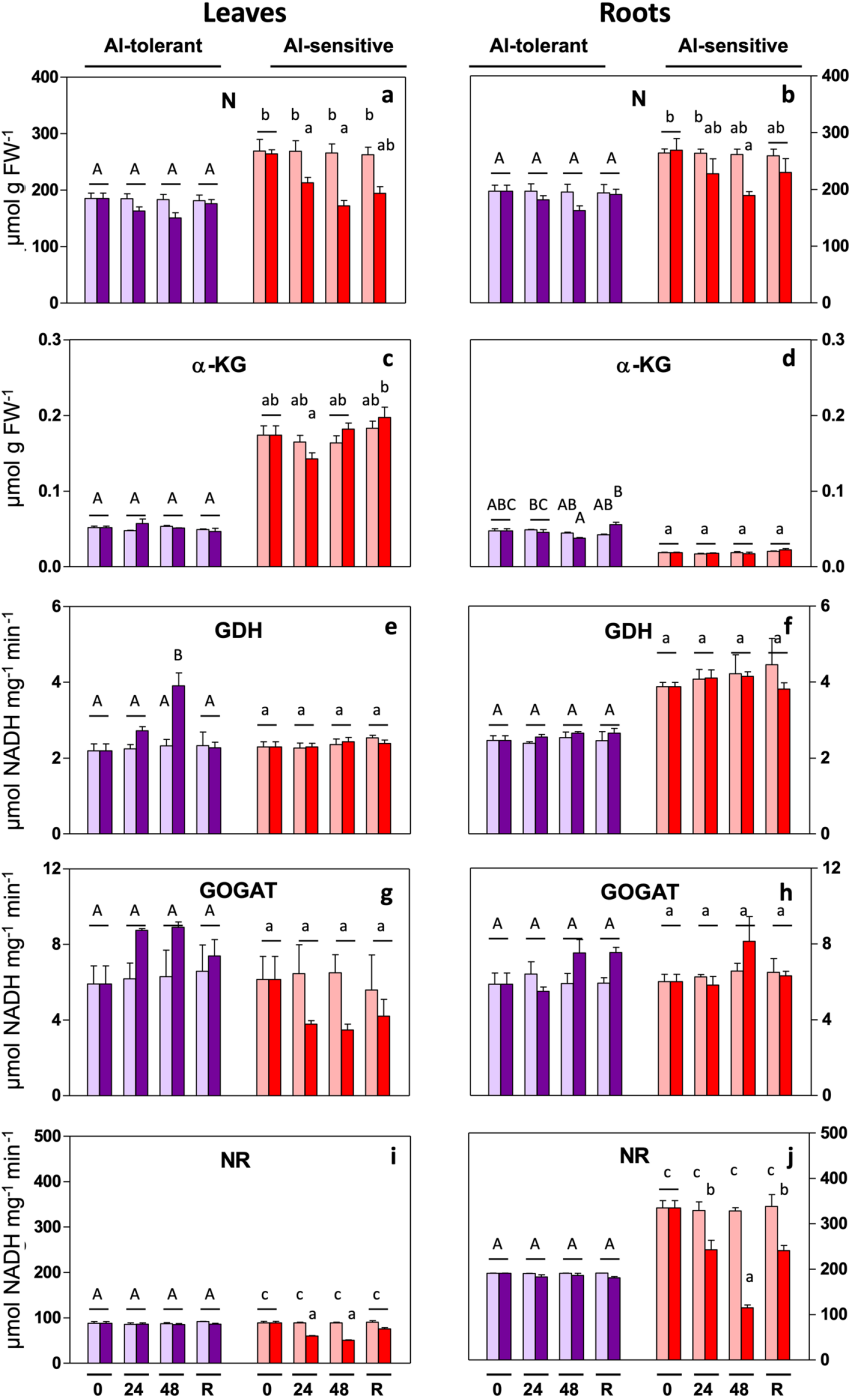
Al-induced changes in N-metabolism in rye seedlings. Metabolite levels and enzyme activities were determined in leaves and roots of Al-tolerant (Beira) and Al-sensitive (RioDeva) genotypes after 24 h and 48 h of exposure and 48 h of recovery. Bars: purple: genotype Beira; red: genotype RioDeva; light color: control (no Al); dark color: 5 mg L^−1^ Al. Panels: (**a**,**b**): nitrogen (N); (**c**,**d**): α-ketoglutarate (α-KG); (**e**,**f**): glutamate dehydrogenase (GDH); (**g**,**h**): glutamine oxoglutarate aminotransferase (GOGAT); (**i**,**j**): nitrate reductase (NR). The results are the mean of 3 or 4 experiments (SD, see Methods). Letters indicate a significant difference at *P* < 0.05 (capital letters: tolerant line; small letters: sensitive line).

Amino acid levels in plants are tightly correlated with N metabolism. Therefore in the frame of understanding the Al-induced Pro changes, we investigated metabolites (N, α-KG, Gln, Glu) and key enzymes in N fixation (GDH, GOGAT, GS, NR). Nitrogen levels (Fig. 5a) decreased in leaves of the Al-sensitive line. Also total protein content decreased (supplementary table 1a, b), Gln and Glu changed as described above, and α-KG remained unchanged in leaves and roots (Fig. 5c,d). GOGAT activity did not significantly change upon Al treatment (Fig. 5g,h), but GDH activity showed some increase in leaves in the tolerant line (Fig. 5e) (description of results on GS activity in a section above). NR activity decreased significantly in each organ in the Al-sensitive line (Fig. 5i,j).

## Discussion

In recent years, Pro has been demonstrated to be much more versatile than being simply a proteinogenic amino acid. In plants, Pro levels increase in response to a variety of stresses and elevated Pro probably provides multiple-types of protection. Additionally, metal exposure is indicated to alter Pro levels, suggesting the involvement of Pro in the defence against metal induced damage^1^. High Pro levels have been suggested as a primary source of Al tolerance in maize, tea and buckwheat^14–16^. In addition, in date palm, mung bean and spring wheat, exogenous application of Pro demonstrated its protective effect against metal toxicity^17–19^.

Despite the importance of Pro in stress tolerance, the detailed biosynthetic changes leading to altered Pro levels are rarely characterized. Nevertheless, exploring Pro as a target for stress tolerance improvement could benefit from such knowledge. We, therefore, analysed Pro biosynthesis in the roots and leaves of Al-sensitive (RioDeva) and Al-tolerant (Beira) rye seedlings, after short-term Al-exposure. The effects of similar Al treatments on growth and oxidative stress parameters in these genotypes have previously been published^13^.

The results show that Al-treatment increased Pro concentrations, in the roots and leaves of each genotype, although to different extents (see below). In the Al-tolerant rye line, increases were about threefold, whereas increases of only about 20% were observed in the Al-sensitive line. Pro levels, and the Pro increases induced by Al are similar as in previous works e.g*.*^17^. Pro levels returned considerably after stress removal and often to near-normal levels. These findings are consistent with responses to metal toxicity in other species and support the role of Pro in Al tolerance. It should be kept in mind that Al exposure also affects the plant water status, as reflected in reduced stomatal conductance^20^. Water deficiency is known to induce Pro levels, in particular in roots^1^. Therefore, the Pro increases we observed could in part originate from such water status effect.

To understand the molecular basis of the Pro increases, we compared changes in Pro metabolism pathways between genotypes (sensitive vs. tolerant) and between organs (leaves vs. roots).

### Differences in Al responses between an Al-sensitive and Al-tolerant rye genotype

Genotype-specific responses to abiotic stress are observed in numerous species and stress-types, including different sensitivities to metals. In our comparison of responses to Al in a sensitive and tolerant rye line, a first difference in the response we observed, is that although the Glu pathway for Pro synthesis was significantly enhanced in both genotypes upon Al exposure, the enhancement was considerably more pronounced in the the Al-tolerant line. This is for example reflected in more increased P5CS and GS activity. The latter enzyme is considered a regulator of this pathway^4^. Second, ARG activity increased in the tolerant line only, suggesting an extra contribution of Pro through the Orn pathway upon Al stress in this line. Thirdly, ProDH activity decreased in roots and leaves of the Al-tolerant genotype, while no significant changes were observed in the sensitive line. ProDH generally re-oxidizes Pro to P5C, the lower activity may contribute to Pro accumulation (Fig. 1). Inhibition of ProDH also contributes to Pro increases, also in other species and stress conditions^21,22^. ProDH activity returned to near-control levels during recovery, probably contributing to the Pro decrease after stressor removal, except for roots of the Al-tolerant genotype. Interestingly, Pro levels in the absence of Al (control), are higher in the leaves of the Al-sensitive line. Whether this higher Pro makes the Al-sensitive line relatively more tolerant to other stressors has not yet been investigated. It should be noted that upon Al exposure, in absolute numbers, Pro levels in the sensitive line are actually similar to that in the tolerant line, as the result of the higher starting values. This however does not necessarily mean that similar protection levels from Al should be expected as this depends also on the extend of the stress experienced by the organism.

Altogether, our results clearly show there are differences in the response to Al between the sensitive and tolerant rye line at the level of Pro responses. Pro increases in each line, but the mechanism and extent off this induction are different. In each line the Glu pathway activity is increased, but this is more pronounced in the tolerant line. Also, in the tolerant line but not the sensitive line, the Orn pathway for Pro synthesis is activated, and Pro inactivation is down-regulated. These processes are also likely to contribute to the stronger Pro increase in the tolerant line. Additionally, in other studies, Pro has been shown to contribute to genotype-specific tolerance. For example, genotype differences in response to drought, salinity, heat, and cold have been associated with differences in Pro levels^23–25^. Moreover, differential accumulation of Pro is responsible for the genotype-specific differences in tolerance to Cu, Ni, and Cr, in soybean, *Thlaspi* spp. and rice, respectively. Therefore, our findings are consistent with these results. This suggests that responses in Pro metabolism may be a more common factor contributing to differences between genotypes, in multiple species and across multiple stresses.

### Differences in Al responses in leaves and roots

Stress responses likely vary between organs, so we investigated whether Al-induced Pro increases in leaves and roots, in each genotype, originated from the same or different pathways. First, in non-stressed plants, Pro levels were comparable in leaves and roots of the tolerant line, but higher in leaves than in roots in the sensitive line. The observation that Pro levels are similar in roots of the Al-tolerant and -sensitive line, is noteworthy as the roots are the primary sensing location for Al toxicity. This suggests that at least the basic levels of Pro in the rye root are not an important factor in the Al-sensitivity differences.

In response to Al, we observed that in roots and leaves and in each line, Pro accumulation occurs primarily from the upregulation of the Glu pathway. However, in roots, but not in leaves, of the tolerant line, OAT activity is increased, possibly contributing to Orn-dependent Pro synthesis. These results are consistent with studies in which elevated Pro synthesis under stress is ascribed to up-regulation of the Glu pathway, through the increase of P5CS^5,26^. However, in some instances, also the Orn pathway has been shown to contribute considerably to Pro biosynthesis, partially depending on the severity of stress^22,27^. Activation of the Orn pathway may also be correlated to N-levels (see below). Activation of both Glu and Orn pathways results in higher Pro levels in the roots of the tolerant genotype. As the roots are the primary target of Al toxicity, activation of both pathways might be essential for this organ to cope with Al stress. Differences in Pro accumulation in roots and shoots, in response to metals, have previously been reported. For example, Pro levels were higher in roots in various species exposed to Cu, Pb, Cd or Hg^28–32^. However, in other species, higher Pro levels in shoots have been reported than in roots (see review by Sharma and Dietz^33^).

Al effects in leaves necessarily result directly from Al taken up and transported to leaf cells, or indirectly from systemic stress-signal effects that are translocated from the roots. There is some controversy as to which effect predominates, as in some studies leaf Al levels were effectively increased upon Al exposure^34,35^, whereas no increases were found in the leaves in other studies^36^. Our own determinations show increased Al levels in the leaves, supporting the possibility of direct interaction of Al with leaf cell metabolism (supplementary table 1A).

### Relation of Al-induced Pro changes to N metabolism

Amino acid metabolism is closely connected to N metabolism. Nitrogen content and NR activity decreased under Al stress in leaves and roots of the sensitive genotype but much less in the tolerant line. This raises the question of whether and how N assimilation is related to the differences in Pro changes between the two phenotypes. It is tempting to speculate that reduced N assimilation in the sensitive line, limits the ability to further elevate Pro levels, therefore reducing tolerance. Very few studies have simultaneously determined changes in Pro synthesis and N assimilation. In those studies, elevated N levels resulted in elevated Pro^37,38^. Moreover, the N-related elevated Pro appears to originate from the Orn pathway^37^, supporting our speculation, as this pathway likely provides the extra Pro increase in the tolerant line. In addition, we recently demonstrated that Pro increases in grasses and legumes under drought stress occur predominantly through the Glu pathway in grasses, and the Orn pathway in legumes^4^. This difference may well be related to the better N-status in legumes. If this view on N-status and Orn pathway-activation is correct, one could imagine that the higher Al tolerance in one rye genotype may indeed be related to a more stable N-assimilation and N content. This would argue for a causal role of N-levels/fixation in controlling Pro biosynthesis pathways. However, alternatively, less-effective Pro supply and reduced N-assimilation in the sensitive line may also be parallel consequences of a common upstream genotypic difference in the Al response. Obviously more testing of these interpretations is necessary.

## Conclusion

In summary, we propose that a stronger stress-induced increase in Pro accumulation enhances tolerance against Al in one of two tested rye genotypes. Moreover, this genotype difference is related to activation of the Orn-biosynthetic pathway, and a decrease in Pro-inactivation activity. We also observed differences in the response in leaves and roots, as the Orn pathway appears to be involved only in roots. Together this indicates that Pro levels are controlled by multiple factors. The correlation between Pro changes and differences in Al-sensitivity between genotypes, supports a role for Pro in Al tolerance. However, the demonstration of Pro contributing to the differences in Al tolerance between the genotypes, does not by itself explain by which molecular mechanism(s) this occurs. Our results also provide support to the idea that differential responses in Pro-biosynthesis pathways are linked to N assimilation. The observations that differential effects on Pro-biosynthesis also occurs also in other species, as well as the fact that Pro has previously been indicated as a genotype-specific factor in plant responses, makes us believe that the mechanisms elucidated in the Al-exposure of rye seedlings are a more common stress-protection mechanism than previously considered.

## Methods

### Experimental setup

Two rye (*Secale cereale* L.) genotypes differing in Al tolerance (Al-tolerant: var. Beira and Al-sensitive: var. RioDeva) were selected^12^. Rye seeds were sterilized by 10 min treatment with sodium hypochlorite (5% w/v), and hydroponically grown in a modified Hoagland solution as described^13^, in a growth cabinet at 25 °C, 16/8 L/D photoperiod, PAR at 60 µmol m^−2^ s^−1^. The nutrient solution was continuously aerated and the pH was maintained at 4.0, by daily checking and adjusting with dilute HCl or NaOH). Leaves and roots were harvested from 2 day-old seedlings, immediately before Al treatment. Samples were weighed, immediately frozen in liquid nitrogen, ground to a powder, and stored at − 80 °C. Aluminum was supplied as AlCl_3_⋅6H_2_O to obtain 5 mg L^−1^ (185 μM) of Al. Al-exposed leaves and roots were harvested after 24 and 48 h exposure. At 48 h, the Hoagland solution was replaced with Al-free medium and leaves and roots were harvested after 48 h (recovery). Each experimental treatment was performed 4 times. In each experiment all enzyme activities were determined (4 repetitions) and in 3 experiments also metabolites were determined (3 repetitions). Measurements were always performed with 3 replicates.

### Metabolite determination

Leaves or roots (100 mg) were homogenized (MagNALyser, Roche, Vilvoorde, Belgium; 1 min, 7000 rpm) in 1 mL of 80% (v/v) aqueous ethanol and spiked with norvaline as an internal control, and centrifuged at 20,000* g* for 20 min. Sample preparation, metabolite (amino acids and α-ketoglutarate, α-KG) extraction and quantification were performed as described previously^4,39^. In brief, the extract supernatant was vacuum dried and resuspended in chloroform. This suspension was mixed with a water re-extraction of the plant residue and after separation the aqueous phase was collected for amino acid quantification by UPLC (UPLC-tqd, Milford, MA, USA). Metabolite concentrations are expressed on a fresh weight (FW) basis. We found that expressing on FW or dry weight made no qualitative difference in the results. This is consistent with the lack of differential effects of Al on the water status of the rye seedlings (supplementary table 1A). Results are expressed as the mean ± SD (standard deviation) in all figures and supplementary table 1A, B.

### Enzyme activity determination

Enzyme activities were determined according to published procedures, without changes. For semi-high throughput processing of samples, a microplate reader was used (Synergy Mx; Biotek Instruments Inc., Winooski, VT, USA), and assay volumes were reduced. Assay conditions (protein concentration, time) were adjusted to obtain linear rates. The measuring principle for each enzyme was as follows: (1) glutamine synthetase (GS, EC 6.3.1.2), monitoring of γ‐glutamyl hydroxamate (γGH) (*A*_500_); (2) pyrroline-5-carboxylate reductase (P5CR, EC 1.5.1.2), monitoring P5C-dependent NADH oxidation (*A*_340_)^4^; (3) pyrroline-5-carboxylate synthase (P5CS, EC 2.7.2.11/1.2.1.41), accumulation of γ‐glutamyl hydroxamate (*A*_535_)^4^; (4) proline dehydrogenase (ProDH, EC 1.5.99.8), Pro-dependent reduction of 2,6-dichloroindophenol (DCIP, *A*_600_)^4^; (5) arginase (ARG, EC 3.5.3.1), measuring urea production using diacetyl monoxime (*A*_465_)^4^; (6) ornithine aminotransferase (OAT, EC 2.6.1.13), reduction of NADH (A_340_)^4^; (7) pyrroline-5-carboxylate dehydrogenase (P5CDH, EC 1.2.1.88) by oxidation of NADH (A_340_); (8) glutamine oxoglutarate aminotransferase (GOGAT, EC 2.6.1.53) determining glutamine-dependent NADH oxidation (A_340_)^40^; (9) glutamate dehydrogenase (GDH, EC1.4.1.2), determining 2-oxoglutarate-dependent NADH oxidation (A_340_)^40^; and (10) nitrate reductase (NR, EC 1.7.1.1), measuring nitrite-dependent NADH oxidation (A_340_)^41^. Protein concentrations were determined according to Lowry et al*.*^42^, using bovine serum albumin (BSA) as the standard. Results are expressed as the mean ± SD (standard deviation) in the figures and supplementary table 1A, B.

### Statistical analysis

Results were expressed as mean ± SD (standard deviation) and analyzed by three-way ANOVA (SPSS Statistica 23, SPSS Inc., Chicago, IL, USA), with genotype (G), treatment (T) and organs (O) as fixed variables (ANOVA results in supplementary table 2) as previously described in our previous publications^13,43^. In cases of significant interactions between factors, one-way ANOVA was performed for each factor, and Tukey’s multiple range tests were used to determine significant differences among means. A significance level of *P* < 0.05 was used for rejection of the null hypothesis.

## Supplementary information

Supplementary Information 1.

Supplementary Information 2.

## Data Availability

The datasets generated during and/or analysed during the current study are available from the corresponding author on reasonable request.
