# Supplementary material for: Al exposure increases proline levels by different pathways in an Al-sensitive and an Al-tolerant rye genotype
Source: Sci Rep. 2020 Oct 2;10:16401. doi: 10.1038/s41598-020-73358-9 (PMC7532535; doi:10.1038/s41598-020-73358-9)
Supplement: Supplementary file 1 — Supplementary Information 1. [file 41598_2020_73358_MOESM1_ESM.docx]

**Supplementary table 1A:** Table with of averages and standard deviations for aluminum content, metabolite levels, enzyme activities and water content in *leaves* of rye genotypes.

|  |  | **Leaves** | | | | | | | | | | | | | | | |
| --- | --- | --- | --- | --- | --- | --- | --- | --- | --- | --- | --- | --- | --- | --- | --- | --- | --- |
|  |  | **Al-tolerant** | | | | | | | | **Al-sensitive** | | | | | | | |
|  | **Time** | **0** | | **24** | | **48** | | **96** | | **0** | | **24** | | **48** | | **96** | |
|  | **Al** | **-** | **+** | **-** | **+** | **-** | **+** | **-** | **+** | **-** | **+** | **-** | **+** | **-** | **+** | **-** | **+** |
| **Al** | µg g DW^-1^ | 0.0±0.0 | 0.0±0.0 | 0.0±0.0 | 153±3 | 0.0±0.0 | 164±4 | 0.0±0.0 | 72±4 | 0.0±0.0 | 0.0±0.0 | 0.0±0.0 | 205±8 | 0.0±0.0 | 336±6 | 0.0±0.0 | 153±8 |
| **Arg** | µmol g FW^-1^ | 3.3±0.3 | 3.3±0.3 | 3.4±0.2 | 3.2±0.2 | 3.8±0.2 | 2.90±0.04 | 3.8±0.2 | 3.4±0.1 | 1.2±0.1 | 1.2±0.1 | 1.10±0.02 | 1.2±0.1 | 1.1±0.1 | 1.2±0.04 | 1.1±0.1 | 1.3±0.1 |
| **ARG** | µmol ureum mg^-1^min^-1^ | 3.0±0.1 | 3.0±0.1 | 2.9±0.1 | 2.8±0.3 | 2.8±0.1 | 4.7±0.3 | 2.9±0.1 | 2.8±0.4 | 2.2±0.2 | 2.2±0.2 | 2.2±0.2 | 2.2±0.1 | 2.2±0.1 | 2.3±0.1 | 2.2±0.1 | 2.2±0.2 |
| **GDH** | µmol NADH mg^-1^ min^-1^ | 2.2±0.3 | 2.2±0.3 | 2.3±0.2 | 2.7±0.2 | 2.3±0.2 | 3.9±0.5 | 2.3±0.5 | 2.3±0.2 | 2.3±0.2 | 2.3±0.2 | 2.3±0.2 | 2.3±0.1 | 2.4±0.2 | 2.4±0.2 | 2.5±0.1 | 2.4±0.1 |
| **Gln** | µmol g FW^-1^ | 36±2 | 36±2 | 36±2 | 28±2 | 39±1 | 23±2 | 37±2 | 33±2 | 17±1 | 17±1 | 15±1 | 17±2 | 14±2 | 13±1 | 15±1 | 19±4 |
| **Glu** | µmol g FW^-1^ | 11.2±0.2 | 11±0.2 | 10±1 | 10±0.3 | 11±0.3 | 9.3±1 | 10±1 | 14±1 | 11± 1 | 11±1 | 10± 2 | 10±1 | 10±1 | 8±1 | 10±1 | 10±1 |
| **GOGAT** | µmol NADH mg^-1^ min^-1^ | 6±1 | 6±1 | 6±1 | 9.0±0.1 | 6±2 | 9.0±0.4 | 7±2 | 7±2.0 | 6±2 | 6±2 | 7±2 | 4.0±0.3 | 7±1 | 4.0±0.4 | 6±3 | 4±1 |
| **GS** | µmol 𝛾GH mg^-1^ min^-1^ | 9±1 | 9±1 | 10±1 | 16.0±0.3 | 10±1.0 | 23±4 | 11±2 | 17±1 | 7±1 | 7±1 | 8±1 | 7.0±0.1 | 8±1 | 8.0±0.2 | 7±1 | 8±1 |
| **N** | µmol g FW^-1^ | 185±14 | 185±14 | 185±12 | 163±11 | 183±13 | 151±13 | 181±14 | 176±10 | 269±29 | 269±29 | 269±26 | 213±14 | 266±23 | 172±13 | 263±19 | 194±17 |
| **NR** | mmol NADH mg-1 min^-1^ | 88 ±5 | 88±5 | 86±5 | 86±4 | 88±3 | 85±4 | 92±1 | 86±3 | 90±5 | 90±5 | 89±3 | 60±2 | 89±2 | 57±1 | 91±5 | 76±4 |
| **OAT** | µmol NADH mg^-1^ min^-1^ | 0.90±0.02 | 0.90±0.02 | 0.90±0.01 | 0.80±0.02 | 0.90±0.03 | 0.80±0.02 | 0.900±0.005 | 0.80±0.01 | 0.90±0.02 | 0.90±0.02 | 0.90±0.02 | 0.70±0.03 | 0.90±0.01 | 0.05±0.02 | 0.90±0.04 | 0.70±0.03 |
| **Orn** | µmol g FW^-1^ | 18±3 | 18±3 | 30±3 | 20±2 | 28±7 | 15± 2 | 23±6 | 15±1 | 8±1 | 8±1 | 7±1 | 7±1 | 7±1 | 7±1 | 8±1 | 9±1 |
| **P5C** | µmol g FW^-1^ | 1.70±0.02 | 1.70±0.02 | 1.7±0.2 | 1.1±0.1 | 1.8±0.2 | 1.3±0.2 | 1.6±0.2 | 1.5±0.2 | 1.6±0.2 | 1.6±0.2 | 1.5±0.1 | 0.8±0.1 | 1.4±0.1 | 0.6±0.03 | 1.5±0.2 | 0.9±0.1 |
| **P5CDH** | µmol NADH mg^-1^ min^-1^ | 3.7±0.3 | 3.7±0.3 | 4±1 | 3.5±0.2 | 4±1 | 2.4±0.4 | 4±1 | 3.9±0.4 | 5±1 | 5±1 | 4.4±0.4 | 4.4±0.2 | 4±1.0 | 4.8±0.2 | 5±1 | 4.1±0.1 |
| **P5CR** | µmol NADH mg^-1^ min^-1^ | 2.80±0.04 | 2.80±0.04 | 3.10±0.04 | 3.6±0.2 | 2.8±0.1 | 6.0±0.2 | 2.9±0.1 | 3±1 | 1.2±0.1 | 1.2±0.1 | 1.4±0.2 | 1.2±0.1 | 1.4±0.2 | 1.4±0.2 | 1.4±0.2 | 1.4±0.2 |
| **P5CS** | µmol NADH mg^-1^ min^-1^ | 3.30±0.04 | 3.3±0.1 | 3.5±0.1 | 6.2±0.3 | 3.3±0.1 | 8.6±0.3 | 3.50±0.04 | 5±1 | 2.4±0.2 | 2.4±0.2 | 2.30±0.03 | 27±1 | 2.5±0.2 | 4.4±0.6 | 2.4±0.2 | 2.6±0.1 |
| **Pro** | µmol g FW^-1^ | 5.2±0.3 | 5.2±0.3 | 6.5±0.3 | 9.4±1.0 | 6.1±0.3 | 17±1 | 5.3±0.3 | 12±1 | 13±1 | 13±1 | 11±1 | 14±2 | 11±1 | 16±1 | 11±1 | 13±2 |
| **ProDH** | µmol DCIP mg^-1^ min^-1^ | 0.80±0.04 | 0.80±0.04 | 0.80±0.04 | 0.4±0.1 | 0.8±0.2 | 0.300±0.004 | 0.8±0.2 | 0.7±0.1 | 0.30±0.02 | 0.30±0.02 | 0.30±0.02 | 0.30±0.01 | 0.30±0.04 | 0.2±0.1 | 0.30±0.03 | 0.3±0.1 |
| **Protein** | µmol g FW^-1^ | 40±3 | 40±3 | 36±2 | 33±3 | 36±3 | 30±3 | 36±3 | 35±2 | 53±6 | 53±6 | 53±6 | 35±1 | 52±5 | 29±2 | 52±4 | 39±2 |
| **RWC** | % | 86±1 | 86±1 | 87±1 | 82±1 | 87.0±0.3 | 77±1 | 86±2 | 79±1 | 86±1 | 86±1 | 86±1 | 79±1 | 86±2 | 75.0±0.4 | 88±1 | 88±1 |
| **α-KG** | µmol g FW^-1^ | 0.050± 0.003 | 0.05± 0.003 | 0.050± 0.001 | 0.06± 0.01 | 0.050± 0.001 | 0.050± 0.001 | 0.050± 0.001 | 0.05± 0.01 | 0.17± 0.02 | 0.17± 0.02 | 0.16± 0.01 | 0.14± 0.01 | 0.16± 0.01 | 0.18± 0.01 | 0.18± 0.01 | 0.20± 0.02 |

**Abbreviations:** Al, aluminum; ARG, arginase; GDH, glutamate dehydrogenase; GOGAT, glutamine oxoglutarate aminotransferase; GS, glutamine synthetase; OAT, ornithine aminotransferase; Orn, ornithine; P5C, 1-pyrroline-5-carboxylate; P5CDH, pyrroline-5-carboxylate dehydrogenase; P5CR, pyrroline-5-carboxylate reductase; P5CS, pyrroline-5-carboxylate synthase; ProDH, proline dehydrogenase; RWC, relative water content; α-KG, α-ketoglutarate.

**Supplementary table 1B:** Table with of averages and standard deviations for aluminum content, metabolite levels and enzyme activities in *roots* of rye genotypes.

|  |  | **Roots** | | | | | | | | | | | | | | | |
| --- | --- | --- | --- | --- | --- | --- | --- | --- | --- | --- | --- | --- | --- | --- | --- | --- | --- |
|  |  | **Al-tolerant** | | | | | | | | **Al-sensitive** | | | | | | | |
|  | **Time** | **0** | | **24** | | **48** | | **96** | | **0** | | **24** | | **48** | | **96** | |
|  | **Al** | **-** | **+** | **-** | **+** | **-** | **+** | **-** | **+** | **-** | **+** | **-** | **+** | **-** | **+** | **-** | **+** |
| **Al** | µg g DW^-1^ | 0.0±0.0 | 0.0±0.0 | 0.0±0.0 | 876±21 | 0.0±0.0 | 1176±32 | 0.0±0.0 | 919±32 | 0.0±0.0 | 0.0±0.0 | 0.0±0.0 | 959±13 | 0.0±0.0 | 1344±17 | 0.0±0.0 | 1112±10 |
| **Arg** | µmol g FW^-1^ | 0.60±0.01 | 0.60±0.01 | 0.70±0.02 | 0.70±0.04 | 0.70±0.02 | 0.60±0.004 | 0.70±0.02 | 0.8±0.1 | 1.1±0.1 | 1.1±0.1 | 1.0±0.1 | 1.10±0.03 | 0.8±0.1 | 1.3±0.1 | 0.6±0.1 | 1.3±0.1 |
| **ARG** | µmol ureum mg^-1^min^-1^ | 2.4±0.2 | 2.4±0.2 | 2.4±0.2 | 2.6±0.1 | 2.4±0.1 | 3.4±0.2 | 2.4±0.1 | 2.3 ±0.1 | 2.1±1.1 | 2.1±1.1 | 2.2±0.1 | 2.4±0.1 | 2.10±0.02 | 2.4±0.1 | 2.2±0.2 | 2.4±0.2 |
| **GDH** | µmol NADH mg^-1^ min^-1^ | 2.5±0.2 | 2.5±0.2 | 2.4±0.1 | 2.6±0.1 | 2.5±0.2 | 2.7±0.1 | 2.5±0.3 | 2.7±0.2 | 3.9±0.2 | 3.9±0.2 | 4.1±0.4 | 4.1±0.3 | 4.2±1.0 | 4.2±0.2 | 4.5±1.0 | 3.8 0.2± |
| **Gln** | µmol g FW^-1^ | 9.1±0.5 | 9.1±0.5 | 9.6±0.2 | 8.1±0.3 | 8.8±0.3 | 6±1 | 10±1 | 10±1 | 8±1 | 8±1 | 8±1 | 7±1 | 9±1 | 6.0±0.3 | 10±1 | 9±1 |
| **Glu** | µmol g FW^-1^ | 5.0±0.3 | 5.0±0.3 | 5.2±0.1 | 4.7±0.2 | 4.8±0.2 | 3.6±0.3 | 4.6±0.1 | 5.8 ±0.3 | 5±1 | 5±1 | 5.0±0.4 | 4.9±0.4 | 5.3±0.7 | 4.8±0.4 | 6±1 | 7±1 |
| **GOGAT** | µmol NADH mg^-1^ min^-1^ | 6±1 | 6±1 | 6.4±0.9 | 5.5±0.3 | 5.9±0.7 | 8±1 | 5.9±0.4 | 7.5±0.4 | 6±1 | 6±1 | 6.3±0.2 | 6±1 | 7±1 | 8±2 | 7 ±1 | 6.3± 0.3 |
| **GS** | µmol 𝛾GH mg^-1^ min^-1^ | 5±1 | 5±1 | 6±1 | 6.0±0.2 | 6 ±1 | 9±1 | 5.6±0.2 | 6 ±1 | 14±1 | 14±1 | 14±1 | 14±2 | 12±1 | 22±3 | 13.1±0.3 | 18±1 |
| **N** | µmol g FW^-1^ | 197±15 | 197±15 | 197±18 | 182±11 | 196±19 | 163±12 | 194±21 | 191±12 | 264±11 | 264±11 | 264±10 | 228±37 | 261±14 | 190±10 | 259±1 | 230±35 |
| **NR** | mmol NADH mg-1 min^-1^ | 191±1 | 191±1 | 191±1 | 183±7 | 191±1 | 186±7 | 192±1 | 181±4 | 335±23 | 335±23 | 329±27 | 243±30 | 328±10 | 115±10 | 338±37 | 241±16 |
| **OAT** | µmol NADH mg^-1^ min^-1^ | 3.0±0.1 | 3.0±0.1 | 2.9±0.1 | 2.8±0.3 | 2.8±0.1 | 2.7±0.3 | 2.9±0.1 | 2.8±04 | 2.2±0.2 | 2.2±0.2 | 2.2±0.2 | 2.2±0.1 | 2.2±0.1 | 2.3±0.1 | 2.2±0.1 | 2.2±0.2 |
| **Orn** | µmol g FW^-1^ | 6.3±0.8 | 6.3±0.8 | 6.5±0.1 | 7.2±0.2 | 6.3±0.3 | 5.1±0.1 | 6.4±0.2 | 3.2 ±0.2 | 6.9±0.3 | 6.9±0.3 | 7±1 | 7±1 | 8±1 | 8±1 | 9±1 | 8±1 |
| **P5C** | µmol g FW^-1^ | 0.80±0.04 | 0.80±0.04 | 0.9±0.1 | 0.6±0.1 | 0.80±0.02 | 0.60±0.04 | 0.8±0.1 | 0.7±0.1 | 0.8±0.1 | 0.8±0.1 | 0.8±0.1 | 0.8±0.1 | 0.8±0.1 | 0.06±0.04 | 0.9±0.1 | 1.0±0.2 |
| **P5CDH** | µmol NADH mg^-1^ min^-1^ | 3.7±0.3 | 3.7±0.3 | 4±1 | 3.8±0.1 | 4.0±0.9 | 3.4±0.3 | 4.1±1.2 | 4.8±0.4 | 9±1 | 9±1 | 3±1 | 8±1.7 | 10 ±1 | 7±1 | 9±3 | 10±3 |
| **P5CR** | µmol NADH mg^-1^ min^-1^ | 1.3±1.1 | 1.3±1.1 | 1.3±0.1 | 1.6±0.3 | 1.30±0.03 | 2.4±0.3 | 1.30±0.04 | 1.1±0.2 | 1.30±0.06 | 1.30±0.06 | 1.20±0.04 | 1.4±0.1 | 1.3±0.1 | 3.1±0.4 | 1.3±0.1 | 1.4±0.2 |
| **P5CS** | µmol NADH mg^-1^ min^-1^ | 2.80±0.04 | 2.80±0.04 | 3.0±0.1 | 4±1 | 3.40 ±0.04 | 7±1 | 3.3±0.5 | 4±1 | 2.6±0.4 | 2.6±0.4 | 3±1 | 3.0±0.4 | 2.9±0.2 | 4.9±0.4 | 3.0±0.4 | 4.0±0.2 |
| **Pro** | µmol g FW^-1^ | 6.7±0.2 | 6.7±0.2 | 7.2±0.1 | 10.2±0.1 | 6.9±0.2 | 15±2 | 7.2±0.2 | 8.0±0.4 | 6.0 ±0. | 6.0 ±0. | 5.9±0.1 | 6.7±0.4 | 6.4±0.1 | 8.9±0.1 | 6.6±0.1 | 7.3±0.2 |
| **ProDH** | µmol DCIP mg^-1^ min^-1^ | 0.70±0.03 | 0.70±0.03 | 0.8±0.1 | 0.6±0.1 | 0.8±0.1 | 0.50±0.02 | 0.7±0.1 | 1.10±0.04 | 0.40±0.02 | 0.40±0.02 | 0.4±0.1 | 0.50±0.02 | 0.4± 0.1 | 0.30±0.03 | 0.4±0.1 | 0.50±0.01 |
| **Protein** | µmol g FW^-1^ | 39±23 | 39±23 | 39±4 | 34±2 | 38±4 | 30±1 | 38±4 | 37±2 | 52±2 | 52±2 | 52±2 | 39±3 | 51±3 | 31±3 | 51±3 | 42±2 |
| **α-KG** | µmol g FW^-1^ | 0.050± 0.004 | 0.050± 0.004 | 0.050± 0.001 | 0.05± 0.01 | 0.050± 0.002 | 0.040± 0.001 | 0.040± 0.001 | 0.06± 0.01 | 0.020± 0.001 | 0.020± 0.001 | 0.020± 0.001 | 0.020± 0.001 | 0.020± 0.001 | 0.020± 0.003 | 0.020± 0.001 | 0.02± 0.002 |

Abbreviations see above.
