# Supplementary material for: Al exposure increases proline levels by different pathways in an Al-sensitive and an Al-tolerant rye genotype
Source: Sci Rep. 2020 Oct 2;10:16401. doi: 10.1038/s41598-020-73358-9 (PMC7532535; doi:10.1038/s41598-020-73358-9)
Supplement: Supplementary file 2 — Supplementary Information 2. [file 41598_2020_73358_MOESM2_ESM.docx]

**Supplementary table 2:** Table with outcome (P-values) of three-way ANOVA analysis. Italicized numbers are non-significant (P>0.05).

|  | **Genotype (G)** | **Treatment (T)** | **Organ (O)** | **G x T** | **G x O** | **T x O** | **G x T x O** |
| --- | --- | --- | --- | --- | --- | --- | --- |
| **Al** | 0.000 | 0.000 | 0.000 | 0.000 | 0.000 | 0.000 | 0.000 |
| **Arg** | 0.001 | 0.000 | 0.000 | 0.008 | 0.000 | 0.000 | 0.007 |
| **ARG** | 0.000 | 0.000 | 0.014 | 0.000 | 0.050 | *0.075* | 0.015 |
| **GDH** | 0.000 | 0.001 | 0.000 | 0.001 | 0.033 | 0.000 | 0.000 |
| **Gln** | 0.000 | 0.000 | 0.000 | 0.047 | 0.000 | 0.008 | 0.028 |
| **Glu** | 0.000 | 0.000 | 0.000 | 0.004 | 0.050 | 0.007 | 0.024 |
| **GOGAT** | 0.000 | 0.007 | 0.000 | 0.023 | 0.000 | 0.039 | 0.003 |
| **GS** | 0.001 | 0.000 | 0.000 | 0.023 | 0.000 | 0.042 | 0.000 |
| **N** | 0.000 | 0.000 | 0.012 | 0.000 | 0.014 | 0.000 | 0.012 |
| **NR** | 0.000 | 0.000 | 0.018 | 0.036 | *0.078* | 0.034 | 0.042 |
| **OAT** | 0.000 | 0.000 | 0.000 | 0.000 | 0.000 | 0.000 | 0.000 |
| **Orn** | 0.000 | 0.000 | 0.041 | 0.000 | 0.000 | 0.049 | 0.033 |
| **P5C** | 0.000 | 0.000 | 0.000 | 0.000 | 0.018 | 0.020 | 0.027 |
| **P5CDH** | 0.000 | 0.008 | 0.000 | 0.031 | 0.000 | *0.091* | 0.041 |
| **P5CR** | 0.000 | 0.000 | 0.000 | 0.000 | 0.000 | 0.037 | 0.000 |
| **P5CS** | 0.000 | 0.000 | 0.044 | 0.000 | 0.000 | 0.039 | 0.034 |
| **Pro** | 0.000 | 0.000 | 0.000 | 0.000 | 0.000 | 0.000 | 0.001 |
| **ProDH** | 0.000 | 0.000 | 0.000 | 0.000 | 0.030 | 0.002 | 0.000 |
| **Protein** | 0.000 | 0.000 | 0.002 | 0.043 | 0.020 | 0.002 | 0.041 |
| **α-KG** | 0.000 | 0.014 | 0.000 | 0.006 | 0.000 | 0.011 | 0.003 |

**Abbreviations:** ARG, arginase; GDH, glutamate dehydrogenase; GOGAT, glutamine oxoglutarate aminotransferase; GS, glutamine synthetase; OAT, ornithine aminotransferase; Orn, ornithine; P5C, 1-pyrroline-5-carboxylate; P5CDH, pyrroline-5-carboxylate dehydrogenase; P5CR, pyrroline-5-carboxylate reductase; P5CS, pyrroline-5-carboxylate synthase; ProDH, proline dehydrogenase; α-KG, α-ketoglutarate. (RWC not included in ANOVA since no root data available).
